# Auditory Attention and Spatial Unmasking in Children With Cochlear Implants

**DOI:** 10.1177/2331216520946983

**Published:** 2020-08-19

**Authors:** Sara M. Misurelli, Matthew J. Goupell, Emily A. Burg, Rachael Jocewicz, Alan Kan, Ruth Y. Litovsky

**Affiliations:** 1 Waisman Center, University of Wisconsin-Madison; 2 Department of Surgery, Division of Otolaryngology, University of Wisconsin School of Medicine and Public Health; 3 Department of Hearing and Speech Sciences, University of Maryland; 4 School of Engineering, Macquarie University, Sydney, Australia

**Keywords:** cochlear implant, auditory attention, children, spatial

## Abstract

The ability to attend to target speech in background noise is an important skill, particularly for children who spend many hours in noisy environments. Intelligibility improves as a result of spatial or binaural unmasking in the free-field for normal-hearing children; however, children who use bilateral cochlear implants (BiCIs) demonstrate little benefit in similar situations. It was hypothesized that poor auditory attention abilities might explain the lack of unmasking observed in children with BiCIs. Target and interferer speech stimuli were presented to either or both ears of BiCI participants via their clinical processors. Speech reception thresholds remained low when the target and interferer were in opposite ears, but they did not show binaural unmasking when the interferer was presented to both ears and the target only to one ear. These results demonstrate that, in the most extreme cases of stimulus separation, children with BiCIs can ignore an interferer and attend to target speech, but there is weak or absent binaural unmasking. It appears that children with BiCIs mostly experience poor encoding of binaural cues rather than deficits in ability to selectively attend to target speech.

## Introduction

This work seeks to understand the mechanisms related to children’s ability to hear and understand speech in a noisy room, and to attend to and engage in conversation with one person while ignoring others who are talking in the background. Binaural hearing and selective auditory attention impact an individual’s ability to successfully function in noisy environments. They are especially important for children because they spend a great deal of time in noisy environments such as classrooms, playgrounds, and cafeterias, where successful communication is critical for their educational and social development. This work attempts to differentiate between some of the peripheral and central factors affecting unmasking of speech for children who listen with bilateral cochlear implants (BiCIs).

Masking of speech sounds can be somewhat alleviated by spatially separating the *target* sound source of interest from *interferers* that are either irrelevant or interesting sounds, both potentially distracting. The improvement in speech understanding when target and interferers are spatially separated, compared with when they are co-located, is known as spatial release from masking (Kidd et al., 2010) or spatial unmasking (Dirks & Wilson, 1969). Previous work shows that spatial unmasking is demonstrated in normal-hearing (NH) children as young as 2 to 3 years of age (Garadat & Litovsky, 2007; Hess et al., 2018).

Over the past few decades, there has been increasing evidence showing that cochlear implants (CIs) are successful at providing access to sound via electrical stimulation of the auditory nerve to children and adults who are identified with severe to profound sensorineural hearing loss. Many children who receive CIs are placed in mainstream educational settings, which have proven to be beneficial for development of spoken language and aural communication (Langereis & Vermeulen, 2015; Tobey et al., 2004). Standard classrooms, however, typically have unfavorable listening conditions or target-to-masker ratios (TMRs; Crandell & Smaldino, 2000). Poor TMRs in classrooms can hinder academic performance for all children (Shield & Dockrell, 2008), but especially for those with hearing loss who have more difficulty listening to speech in noise (see Neuman et al., 2012 for a review).

Today, many children who are deaf receive BiCIs, in part, to help overcome difficulties associated with listening in noisy environments. Although multiple studies show improvement on spatial hearing tasks for children with BiCIs versus children with unilateral CIs (Bennett & Litovsky, 2020; Cullington et al., 2017; Galvin et al., 2007; Grieco-Calub & Litovsky, 2010, 2012; Litovsky et al., 2004; Murphy et al., 2011; Reeder et al., 2017; Suneel et al., 2020; Zheng et al., 2015), there are still notable gaps in performance between children with BiCIs and children with NH. When target speech and interfering speech are spatially separated, children with BiCIs typically demonstrate smaller benefits (i.e., spatial unmasking) compared with NH peers who are matched for either chronological age or for number of years of auditory experience (Hess et al., 2018; Misurelli & Litovsky, 2012, 2015). Spatial unmasking in children with BiCIs may be limited, in part, by peripheral encoding deficits resulting in weak or absent transmission of binaural cues through clinically available CI devices (see Kan & Litovsky, 2015 for a review). Other central mechanisms and nondevice-related factors may also contribute to this gap in performance. For example, auditory deprivation and listening to a degraded signal can impact cognitive mechanisms that control attention and memory (Kronenberger et al., 2014) and thus may affect how well listeners are able to attend to a target stimulus in the presence of competing sounds. In addition, interimplant delays may also have an effect. Cullington et al. (2017) found that children with BiCIs who were implanted with the second CI sooner had a larger improvement in speech understanding in noise compared with children who had longer delays between their first and second CIs.

Spatial unmasking of speech stimuli has typically been investigated using testing paradigms that present sounds in the free-field. These paradigms engage both peripheral and central mechanisms in order to successfully decipher target stimuli. Improvements in understanding of target speech stimuli arise from the availability of spatial cues that result from monaural head shadow, binaural summation, and binaural squelch (see Litovsky et al., 2017 for a review). In individuals with NH, sounds arriving at the two ears are processed in the brainstem in the superior olivary complex, providing the ability to take advantage of both binaural and monaural cues for spatial unmasking (Yin et al., 2019). Children with NH can show spatial unmasking similar to adults with NH (Litovsky, 2005; Misurelli & Litovsky, 2012), whereby they benefit from spatial separation when interferers are 90° away from the target and directed toward only one ear. In addition, children with NH also benefit from spatial separation when interferers are located in a symmetrical interferer configuration (e.g., ±90° azimuth with a target at 0° front), where monaural cues are less available, and they must rely mostly on binaural cues for source segregation. In contrast, children with BiCIs show spatial unmasking primarily when interferers are directed toward only one ear (i.e., when they can take advantage of the monaural head shadow). In both children and adults with BiCIs, the benefit of spatial separation of auditory sources is minimal when the monaural head shadow cue is reduced or eliminated by directing interferers to both ears in a symmetrical interferer configuration (Hu et al., 2018; Misurelli & Litovsky, 2015; Rana et al., 2017). It is important to note that the stimuli used during the task, and similarity between targets and interferers play an important role in determining the effect sizes and differences between children and adults (see Litovsky et al., 2017, for a review). A recent review suggests that there are numerous factors, both peripheral and central, that may impact age-related changes in the ability to identify and understand target speech in noise (Leibold & Buss, 2019). For example, Buss et al. (2016) showed that release from masking in children with NH as young as 5 years of age is impacted by the fact that children use context less than adults, rendering top-down repair or restoration of target speech less beneficial.

In contrast to the aforementioned free-field studies where both ears receive sound source information from each location, this study investigated nonspatial factors—specifically selective auditory attention—to provide further insight into weak effects of spatial unmasking observed to date in children with BiCIs. The results of this study may help to better differentiate peripheral versus central factors contributing to the lack of spatial unmasking typical in this population. Performance was thus compared across various ipsilateral (within ear) and contralateral (opposite ear) target-interferer configurations. This testing paradigm was chosen in an attempt to better isolate the ability to selectively attend to target speech in an individual ear (Cherry, 1953). Others have used similar methods in order to investigate auditory attention in children and adults with NH using speech stimuli (Kidd et al., 2008; Wightman & Kistler, 2005; Wightman et al., 2010). Data suggest that the TMR at which adults can identify target speech is lower (i.e., better) than the TMR measured for children, in particular when stimuli are designed to elicit more informational masking (e.g., Wightman & Kistler, 2005). In studies, informational masking is thought to originate from auditory mechanisms beyond the periphery, due to factors that include confusability or uncertainty of the target and interfering sources, even when both remain audible to the listener (Brungart, 2001; Durlach et al., 2003; Kidd & Colburn, 2017). This is different from energetic masking, which has been described using the power spectrum model of masking. Energetic masking occurs when the energy of a masker passes through the same auditory filter as the target (Fletcher, 1940).

Goupell et al. (2016) showed that most adults with BiCIs, who were postlingually deaf and received their CIs during adulthood, were able to ignore an interferer when there was no energetic masking (i.e., with the target in one ear and interferer in the opposite ear). However, speech understanding significantly worsened when the target and interferer were presented to the same ear, and the stimuli elicited energetic masking. In addition, adults with BiCIs from the Goupell et al. (2016) study showed no significant unmasking when the interferer was presented to both ears, in a dichotic condition, contrasting with NH adults who could take advantage of the dichotic stimuli, similar to findings in many other studies (e.g., van Hoesel et al., 2008; but also see the group of adult BiCI participants in Bernstein et al. (2016) as compared with Goupell et al. (2018), who when taken as a single group, showed little binaural unmasking on average). Currently, many adult BiCI users received their CIs as adults and transitioned to hearing with electrical signals either after long-term (e.g., congenital hearing loss or hearing loss in childhood) or short-term (e.g., sudden hearing loss) deafness. Given these previous findings, we were interested in studying the ability of children with BiCIs to perform on similar tasks of auditory attention. Unlike adults, children who are deaf now often receive their CIs at a young age when the auditory pathways are still developing, and their overall listening experience is achieved with electrical hearing.

For this study, we tested children who are implanted with BiCIs using similar conditions to the ones in Goupell et al. (2016). The stimuli delivered to each ear were carefully controlled in order to examine the ability to attend to a target talker while inhibiting interfering speech: in the same ear as the target (Ipsilateral), in the ear opposite the target (Contralateral), or in both ears (Bilateral). By comparing performance in these conditions, the contribution of both peripheral and central influences to unmasking in children with BiCIs can be investigated. Results from this study are compared with results from postlingually deaf adults with BiCIs in the Goupell et al. (2016) to examine effects of age and hearing history on auditory spatial attention.

We hypothesized that children with BiCIs would demonstrate binaural unmasking if they had some acoustic hearing at birth, where there would have been some typical development of the binaural system (Ehlers et al., 2017) and children without acoustic hearing experience would show contralateral speech interference (Bernstein et al., 2016; Goupell et al., 2018). We also hypothesized that, in a task that requires selectively attending to one ear (i.e., attend to the talker in one ear, ignore the separate talker in the other ear), children with BiCIs would be worse than adults with BiCIs because of the generally immature central attention abilities of children versus adults (Best & Miller, 2010).

## Methods

### Participants

Ten children with BiCIs participated in this study. They were between the ages of 10;1 and 17;2 (years;months) with a mean (±standard deviation) age of 14;5 ± 2;0. They all had more than 6 years of bilateral experience. Nine participants were implanted sequentially, and only one received simultaneous BiCIs (CIEH). All nine children who were sequentially implanted received their right implant first. The second/left CI was received somewhere between 10 months to 8 years after their first CI, with an average time between CIs of 2;5 ± 2;7 (see Table I).

**Table 1.** Subject Demographics.

Participant ID	Sex	Chronological age (yr;mo)	Etiology of HL	Bilateral experience (yr;mo)	Age, first CI activation (yr;mo)	Age second CI activation (yr;mo)	Time between first and second CI (yr;mo)	First (right) processor	Second (left) processor
CIEU	F	17;2	Unknown	6;9	4;3	10;5	6;2	N6	N6
CIAY	M	16;4	Unknown	10;4	5;2	5;12	0;10	N6	N6
CIAP	F	16;0	Unknown	10;11	3;6	5;2	1;8	N5	N5
CIBO	F	16;0	EVA/Pendred syndrome	12;1	2;10	3;11	1;1	N5	N5
CIAW	M	15;3	Prenatal CMV exposure	9;9	1;3	5;6	4;3	N5	N5
CIAG	M	14;11	Connexin 26	11;9	1;9	3;1	1;4	N6	N6
CIEV	F	14;2	Unknown	3;2	2;8	10;12	8;4	N5	N5
CIDJ	F	14;1	Hereditary	9;0	1;8	5;1	3;5	N5	N5
CIBI	F	13;8	Mondini dysplasia	10;10	1;1	2;10	1;9	Freedom	Freedom
CIEH	M	10;1	Hereditary	9;0	1;1	1;1	0	N5	N5
Mean		14;5		9;0	2;2	5;0	2;5		
*SD*		2;0		2;6	1;4	3;0	2;7		

*Note.* M = male; F = female; *SD* = standard deviation; HL = hearing loss; EVA = Enlarged vestibular aqueduct; CMV = Cytomegalovirus.

All consent and testing procedures were approved and carried out in accordance with the University of Wisconsin-Madison Health Sciences Institutional Review Board. All children received monetary compensation for their participation.

### Stimuli and Equipment

Stimuli consisted of five-word closed-set matrix sentences, composed in the following format: name, verb, number, adjective, and object (Kidd et al., 2008). Each of the five categories had eight possible words. Target stimuli were spoken by a female talker, and interfering stimuli were spoken by a male talker. Either the target or interferer was presented at 70 dB-A. The level of either the target or interferer was adjusted to achieve the desired TMR. For positive TMRs, the interferer level was reduced. For negative TMRs, the target level was reduced. This was done to minimize the effect of the automatic gain control in the sound processor. Participants were presented stimuli via the direct auxiliary input port of their everyday clinically mapped processors. Testing was conducted in a standard double-walled sound booth.

### Procedure

The experiment included four conditions (Quiet, Contralateral, Ipsilateral, and Bilateral). The Quiet condition had no interferer. The Contralateral condition had an interferer in the ear opposite the target. The Ipsilateral condition had an interferer in the same ear as the target. The Bilateral condition had a diotic interferer presented to both ears. Each participant was tested with a range of TMRs in each condition, whereby TMRs were selected depending on individual performance. TMR was fixed throughout each block, and one block consisted of 10 sentences. Consecutive conditions and TMRs were randomized. In general, we aimed to obtain percentage correct scores at four TMRs per condition per participant, which could be fit with a logistic function to estimate a psychometric function (Wichmann & Hill, 2001). Specifically, we aimed for one TMR near 20% correct, two TMRs around 50% correct, and one TMR near 80% correct. Pilot testing and the data from Goupell et al. (2016) provided a general testing range of TMRs. We then tested that range with one block of trials at each TMR. Depending on performance, we adjusted the range such that, for each subject, we would have four-point psychometric functions that focused mostly on the threshold where the steepest slope occurred and covered most of the performance range. Because of the large number of conditions and limited attention span of the children, we needed to limit the number of points on the psychometric function to only four, which were sufficient for a good estimation of threshold. At least two blocks of TMRs were tested at each desired percentage correct yielding at least 100 words for scoring each TMR (2 blocks × 10 sentences/block × 5 words/sentence). Speech reception thresholds (SRTs) were estimated as the 50% correct point on the psychometric function.

On average, testing lasted approximately 8 hours per participant. Testing was broken down into two to four sessions. Children were given multiple breaks throughout each testing session, and small prizes and snacks were provided in order to keep the participants motivated.

## Results

### Speech Reception Thresholds

**Figure 1.**
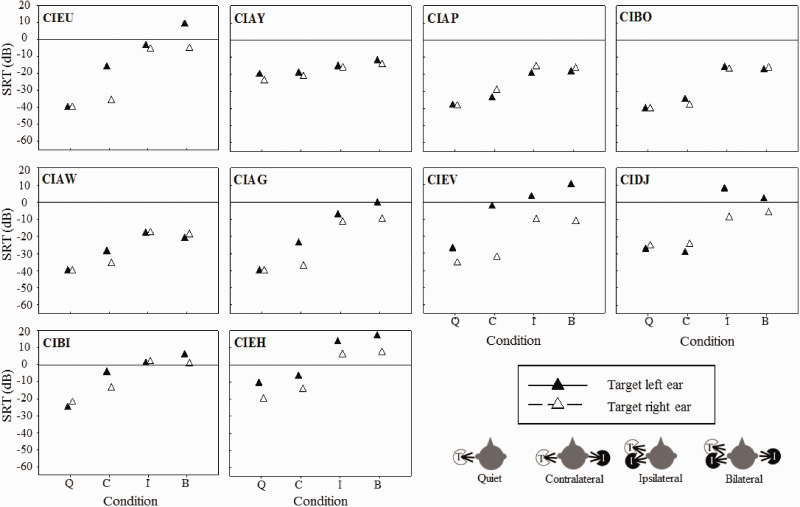
SRTs for each participant and condition. The conditions Q, C, I, B represent Quiet (no interferer), Contralateral (interferer presented to the ear opposite the target), Ipsilateral (interferer presented to the same ear as the target), and Bilateral (interferer in the same and opposite ear to the target), respectively. The filled triangles represent SRTs with the target speech presented to the left ear (odd-numbered), and the open triangles represent SRTs with the target speech presented to the right ear (even-numbered). SRT = speech reception threshold.

Individual children’s SRTs are shown for each condition in Figure 1. In general, the lowest SRTs (best performance) were in the Quiet conditions, when there were no interferers. Performance worsened in all conditions where an interferer was presented. Of the conditions that contained interferers, all individuals demonstrated better performance (lower SRTs) when the interferer was played in the opposite ear (Contralateral) versus the same ear (Ipsilateral) as the target. For some participants, the Bilateral condition elicited lower SRTs than the Ipsilateral condition (difference of at least 3 dB or greater) suggesting that they may have received an unmasking benefit with the introduction of an interferer in the ear opposite the target (e.g., CIAW [3 dB, left ear] and CIDJ [5.9 dB, left ear]). If so, it is possible that this benefit is derived from the participants’ ability to perceive the target and interferer as being spatially separated. A comparison of these conditions showed that some individuals demonstrated very small amounts of unmasking in one or both ears. On average, there was no consistent unmasking in the Bilateral versus the Ipsilateral condition when the target was presented to either ear.

**Figure 2.**
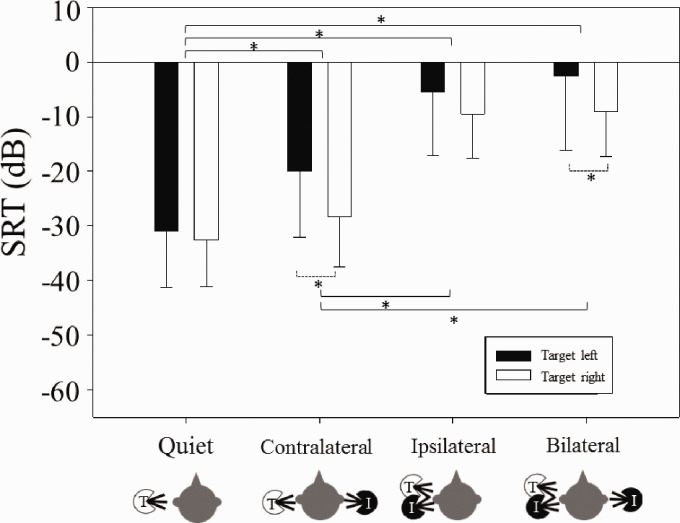
Mean (±*SD*) SRTs for Each Group in Each Condition. Solid and open bars represent SRTs with the target speech presented to the left and right ears respectively. Significant differences are bracketed and indicated with asterisks (*). Solid brackets indicate difference between conditions. Dashed brackets indicate difference within condition between ear (i.e., left versus right). SRT = speech reception threshold.

Group data across conditions are shown in Figure 2. A repeated-measures analysis of variance was conducted to investigate the effects of *condition* (four levels) and *ear* (two levels) on SRT. There was a main effect of *condition*, *F*(3, 27) = 72.9, *p *<* *.001, $\eta_{p}^{2\;}$= 0.89. There was also a main effect of *ear*, *F*(1, 9) = 528.9, *p *=* *.022, $\eta_{p}^{2}$ = 0.46, where the right-ear SRTs were significantly lower than the left-ear SRTs. There was no significant interaction (*p *>* *.05). Post hoc pairwise comparisons (two-sample two-tailed paired *t* tests) using a Bonferroni correction for multiple comparisons were conducted to investigate the difference in SRTs between *conditions* (after collapsing across ears). There was a significant difference in SRTs between all conditions (*p *<* *.001), except when comparing SRTs for the Ipsilateral condition and SRTs for Bilateral condition (*p *>* *.05).

### Selective Auditory Attention Asymmetry

To determine asymmetry between ears in the ability to attend to target speech, selective auditory attention asymmetry was calculated for all individuals. The primary purpose of this calculation was to examine the ability to selectively attend to the target and ignore the interferer in the opposite ear while accounting for baseline sensitivity to the target speech (in quiet). Methods used to calculate asymmetry here were the same methods as that used in Goupell et al. (2016). Asymmetry was defined as the following:

*Asymmetry* = [SRT (Contralateral, target left and interferer right) − SRT (Quiet, target left)] − [SRT (Contralateral, target right and interferer left) – SRT (Quiet, target right)]

**Figure 3.**
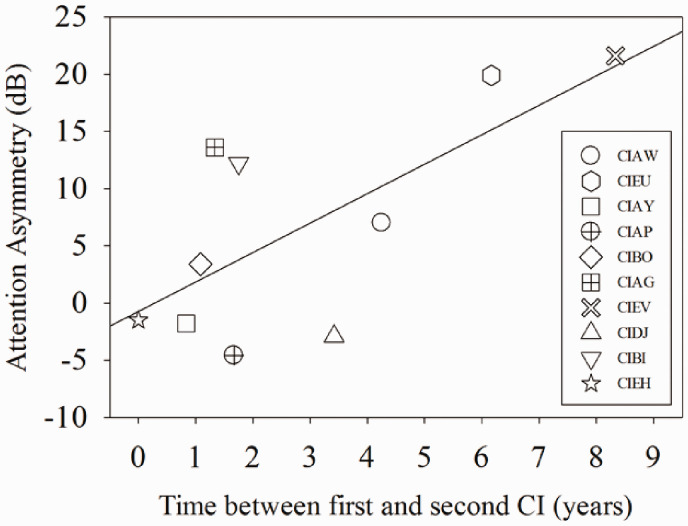
Attention Asymmetry (dB) for Individual Participants. A positive asymmetry indicates reduced ability to ignore an interferer in the right ear (versus left ear). A negative asymmetry indicates reduced ability to ignore an interferer in the left ear (versus right ear). The linear regression shows the relationship between attention asymmetry (dB) and time in years between the activation of the first and second CI. CI = cochlear implant.

Figure 3 shows the attention asymmetry for each child along the vertical axis, as a function of the time between the first and second CI. Results revealed that the two variables were significantly correlated, *r*(8) = .71, *p *=* *.02. Positive asymmetry suggests that the participant had more difficulty ignoring the interferer when it was in the right ear. The magnitude of the asymmetry ranged from 1.8 to 21.6 dB. The largest asymmetries (>10 dB) were always when the interferer was in the right ear, which was the first-implanted ear for all nine of the children who received their CIs sequentially. The two participants with the largest amount of asymmetry were CIEV (asymmetry = 21.6 dB) and CIEU (asymmetry = 19.9 dB).

### Comparison of SRTs Between Children and Adults

**Figure 4.**
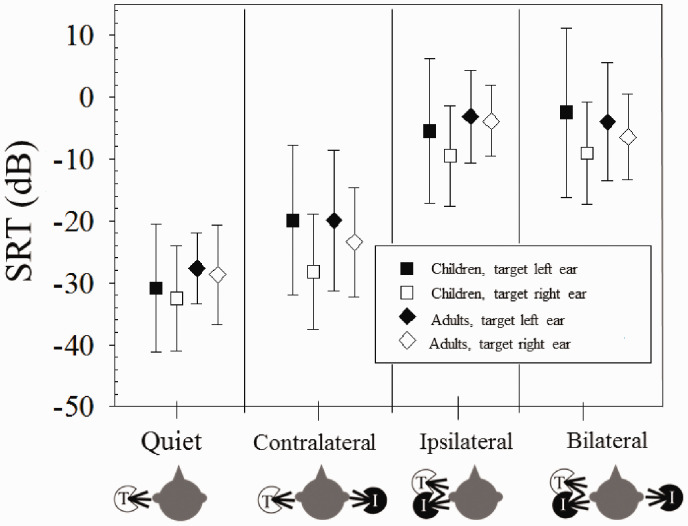
Mean (±1 *SD*) SRTs for BiCI Children and BiCI Adults. The adult data are from Goupell et al. (2016). SRT = speech reception threshold.

It is possible to directly compare the results of this study to Goupell et al. (2016) to understand whether age of implantation is a significant factor in the results. The Goupell et al. study used the same stimuli and tested the same conditions; the main difference was that they tested adults with BiCIs. Figure 4 compares results from this study with published data in adults with BiCIs (see Figure 3 in Goupell et al., 2016). A mixed-design analysis of variance was conducted to investigate within-subject effects of *ear* (left, right) and *condition* (Quiet, Contralateral, Ipsilateral, Bilateral), and between-subject effects of *group* (children, adults). A Greenhouse–Geisser correction was applied for *condition* because the sphericity assumption was violated; post hoc analyses (two-sample two-tailed paired *t* tests) were conducted using a Bonferroni correction. There was a significant main effect of *ear*, *F*(1, 19) = 10.8, *p *=* *.004, $\eta_{p}^{2\;}$= 0.36, where right-ear SRTs were lower than left-ear SRTs. There was also a significant main effect of *condition*, *F*(1.7, 32.5) = 113.7, *p *<* *.0001, $\eta_{p}^{2}\;\;$= 0.86, whereby SRTs in all conditions were significantly different (*p *<* *.001) except for Ipsilateral versus Bilateral (*p *>* *.05). There was a significant *Condition* × *Ear* interaction, *F*(1.7, 33.5) = 4.39, *p *=* *.024, $\eta_{p}^{2}$ = 0.19, likely because the difference in SRTs with target presented to the right versus left ear is not consistent between conditions. For example, the Contralateral, Ipsilateral, and Bilateral conditions show a larger difference between right versus left SRTs than the Quiet condition. There was no effect of *group*, *F*(1, 19) = 0.60, *p *=* *.45, $\eta_{p}^{2\;}$= 0.03, and all other interactions including the factor group were not significant (*p *>* *.05). Note that with 10 child and 11 adult participants, lack of significant interactions may have been a result of too few participants.

## General Discussion

### Contralateral and Spatial Unmasking

Previous work using free-field testing paradigms demonstrated that children with BiCIs typically do not benefit from spatial separation of auditory sources to the same extent as NH children (Ching et al., 2014; Hess et al., 2018; Misurelli & Litovsky, 2012, 2015; Nittrouer et al., 2013; Van Deun et al., 2010). The goal of this study was to investigate the potential influence of selective auditory attention on the diminished spatial unmasking observed in children with BiCIs. The approach used here was the same as that of Goupell et al. (2016), where SRTs were measured in listening conditions designed to elicit unmasking driven by auditory attention being directed to one ear versus the other ear.

Results showed that SRTs in the Quiet condition were significantly better than SRTs in conditions with interferers. In addition, with the interferer in the ear opposite to the target, SRTs were poorer than the quiet condition by about 8 dB. This difference is similar to the finding of Goupell et al. (2016) in adults with BiCIs, which suggests that children with BiCIs were able to ignore an interferer in the opposite ear and attend to the target in a manner that is similar to adults. Masking across ears, however, was not completely eliminated for either of the groups.

Limited dichotic auditory attention data in children with BiCIs have been reported (Koopmann et al., 2020). Unlike spatial release from masking paradigms, whereby sounds reach both ears in free-field and the auditory system is tasked with integrating sounds using binaural mechanisms, in this experiment stimuli delivered to the two ears were controlled such that the Ipsilateral conditions could be compared with the Contralateral conditions. This comparison in children with BiCIs showed approximately 16 dB improvement in SRTs when the interferer was moved from same ear as the target to the opposite ear. This suggests that there is a large amount of unmasking in the most extreme case of separation; without having to integrate information across the ears using binaural processing, the children who participated in this study were able to attend to the target ear and largely ignore the irrelevant information in the ear opposite the target.

To investigate binaural unmasking, we compared the Ipsilateral and Bilateral conditions. The children with BiCIs showed only 2.5 dB of binaural unmasking on average. These results are similar to findings of 1.8 dB for adult BiCI participants in Goupell et al. (2016). The limited amount of unmasking demonstrated here may be due to the poor peripheral encoding of the complex speech signal, which limits the ability to fuse synchronous auditory images to perceive a centrally located interferer. Previous work has investigated binaural unmasking of tones in noise for children with BiCIs. Using research processors to control interaural time differences (ITDs) or interaural level differences (ILDs), electrical signals with single- or multielectrode stimulation have been used to investigate whether children with BiCIs have the ability to use these cues for binaural unmasking of tones in noise (Todd et al., 2016; Van Deun et al., 2011). In Todd et al. (2016), children with BiCIs, on average, were found to be sensitive to binaural cues that aid in binaural unmasking for signal detection with masking level differences of 6.3 dB. However, those findings are limited to situations with carefully controlled binaural stimuli and relatively simple stimuli rather than complex real-world signals such as speech.

With a paradigm not involving binaural unmasking, Ehlers et al. (2017) measured binaural sensitivity to ITDs or ILDs in children with BiCIs using research processors to control binaural cues presented to specified pairs of electrodes in the two cochlear arrays. Sensitivity to ILDs was seen in all children and was similar to that reported in adults. However, ITD sensitivity was poor or absent, except for a few children who had experienced acoustic hearing early in life prior to onset of deafness. That study, along with others investigating similar questions in adults with BiCIs (Litovsky et al., 2010, 2012), suggest that, while ILD circuits in the binaural pathway may be relatively resistant to deprivation or can recover function after participants receive implants in both ears, ITD circuits are more vulnerable to deprivation and might undergo loss of function that cannot be easily recovered. That line of work is consistent with numerous other findings suggesting that ILDs are the primary cues used by individuals with BiCIs for sound localization (Aronoff et al., 2010; Seeber & Fastl, 2008). In this study, ILD cues were available (Bilateral conditions have an infinite ILD) to produce a perceived spatial difference, and yet no unmasking was observed. It may be that ILDs alone are insufficient to provide binaural unmasking of speech (Dieudonné & Francart, 2019; Ihlefeld & Litovsky, 2012). When BiCI users listen in more realistic environments using clinical speech processors, the binaural cues are not preserved with fidelity, which is likely to be one of the reasons that binaural unmasking is small (Kan & Litovsky, 2015). Future work is needed to investigate how sensitivity to binaural cues in these controlled environments could lead to benefit BiCI users in unmasking of speech in more realistic environments.

### Selective Auditory Attention Asymmetry

Selective auditory attention asymmetry was computed from the SRTs; positive values indicate that the individual demonstrated reduced ability to ignore an interferer opposite the target when the interferer was in the right versus the left ear. Our data revealed that the children with BiCIs showed a range of asymmetry (see Figure 3). Furthermore, children with BiCIs who showed large amounts of asymmetry showed positive (right ear directed), rather than negative (left ear directed), asymmetry. Interestingly, all children who were implanted sequentially received their CI in the right ear first. Therefore, our results suggest that children with CIs are better able to attend to a target talker and ignore a contralateral interferer when the target is on the side of their first CI. Previous free-field studies have shown that children with BiCIs demonstrate more spatial release from masking when the target is played at 0° azimuth and the interferer is directed toward the side of the second CI, rather than the side of the first CI (Litovsky et al., 2006; Mok et al., 2007; Peters et al., 2007; Van Deun et al., 2010). In other words, this study together with previous research in the free-field suggests that the interferer is more difficult to ignore when it is directed toward the first CI. Our results also showed a significant relationship between asymmetry and time between the first and second CI (Figure 3); the two individuals with BiCIs who demonstrated the most asymmetry (CIEV and CIEU) are also the two children who had the most time between the activation of the first and second CI. Recent work in adults with BiCIs suggests that individuals who had a longer time between activation of their first and second CI, or who experienced a long period of unilateral hearing, had the largest asymmetry between ears, whereby one ear became significantly more dominant than the other (Goupell et al., 2018). Research in animal models of BiCIs (e.g., Kral et al., 2013) and children with BiCIs (Gordon et al., 2013) have suggested that early auditory deprivation in one ear, and prolonged stimulation in the other ear, results in an aural dominance that is perhaps irreversible. Consequently, studies have found that children who receive both CIs within approximately 1.5 to 4 years are more likely to have symmetrical development of the auditory pathways (Gordon et al., 2013, 2015; Illg et al., 2019). The correlation between attention asymmetry and years between the first and second CI found in this study is consistent with the neurophysiological data, suggesting that benefits obtained from BiCIs are at least partially determined by the time between CIs (see Litovsky & Gordon, 2016 for a review). In addition, there is also some evidence to suggest that the ability to attend to speech in the left ear improves in childhood (Geffen, 1978). Therefore, it may be that at a young age of 7 years the right-ear advantage for speech stimuli in children was more pronounced, and the ability to attend to speech equally in the left ear was still developing.

Comparing SRTs between children with BiCIs and adults with BiCIs from the Goupell et al. (2016) study revealed no developmental effects; SRTs did not significantly differ between children and adults (Figure 4). Note that the limited samples size in both the child (*n* = 10) and adult (*n* = 11) groups, as well as the range of ages for the children (up to 14;5 years), may have obscured developmental effects. A plethora of studies on auditory development, where children listen to speech in noise and use various cues for release from masking suggest that developmental effects exist, but only under some conditions, in particular when the stimuli and tasks are complex or when there is uncertainty as to what is to be ignored when an interferer is present (Litovsky, 2015).

Finally, it should be noted that these data just begin to separate the effects of peripheral versus central processing effects on spatial hearing and auditory attention. Future work could consider objective measures to better separate peripheral and central effects (electrically evoked compound action potentials and brainstem or cortical evoked potential measurements, respectively). Accounting for more cognitive attention effects would be worthwhile, as would relating the effects measured in this study to those using simpler stimuli like central interference phenomenon or contralateral masking (Lin et al., 2013).

## Summary

This study measured binaural unmasking and selective auditory attention in children with BiCIs. We found that children with BiCIs were able to ignore an interferer in the ear opposite to the target, similar to the postlingually deafened adults with BiCIs (Goupell et al., 2016). In addition, the children with BiCIs did not demonstrate binaural unmasking when the interferer was presented to both ears, similar to what occurred in adults with BiCIs (Goupell et al., 2016). When comparing the SRTs between the child and adult groups, there were no significant differences in any of the conditions. This suggests little role for development in binaural unmasking and selective auditory attention. In addition, we found that children had a more difficult time ignoring an interferer when it was directed to first CI, and that this difficulty increased with an increase in delay between the first and second CI. Similar to previous work, this argues that shorter interimplant intervals may be desirable.
